# The tyrosine phosphatase SHP2 controls TGFβ-induced STAT3 signaling to regulate fibroblast activation and fibrosis

**DOI:** 10.1038/s41467-018-05768-3

**Published:** 2018-08-14

**Authors:** Ariella Zehender, Jingang Huang, Andrea-Hermina Györfi, Alexandru-Emil Matei, Thuong Trinh-Minh, Xiaohan Xu, Yi-Nan Li, Chih-Wei Chen, Jianping Lin, Clara Dees, Christian Beyer, Kolja Gelse, Zhong-Yin Zhang, Christina Bergmann, Andreas Ramming, Walter Birchmeier, Oliver Distler, Georg Schett, Jörg H. W. Distler

**Affiliations:** 1 Department of Internal Medicine 3–Rheumatology and Immunology, Friedrich-Alexander-University Erlangen-Nürnberg (FAU) and University Hospital Erlangen, Ulmenweg 18, 91054 Erlangen, Germany; 2 Department of Medicinal Chemistry and Molecular Pharmacology, Purdue University, 575 Stadium Mall Drive Indiana, West Lafayette, 47907 USA; 3 Department of Trauma Surgery, Friedrich-Alexander-University Erlangen-Nürnberg (FAU), Krankenhausstraße 12, 91054 Erlangen, Germany; 4 Max Delbrück Center for Molecular Medicine (MDC), Robert-Rössle-Str. 10, 13092 Berlin, Germany; 5 Department of Rheumatology, University Hospital Zurich, Gloriastrasse 25, 8091 Zurich, Switzerland

## Abstract

Uncontrolled activation of TGFβ signaling is a common denominator of fibrotic tissue remodeling. Here we characterize the tyrosine phosphatase SHP2 as a molecular checkpoint for TGFβ-induced JAK2/STAT3 signaling and as a potential target for the treatment of fibrosis. TGFβ stimulates the phosphatase activity of SHP2, although this effect is in part counterbalanced by inhibitory effects on SHP2 expression. Stimulation with TGFβ promotes recruitment of SHP2 to JAK2 in fibroblasts with subsequent dephosphorylation of JAK2 at Y570 and activation of STAT3. The effects of SHP2 on STAT3 activation translate into major regulatory effects of SHP2 on fibroblast activation and tissue fibrosis. Genetic or pharmacologic inactivation of SHP2 promotes accumulation of JAK2 phosphorylated at Y570, reduces JAK2/STAT3 signaling, inhibits TGFβ-induced fibroblast activation and ameliorates dermal and pulmonary fibrosis. Given the availability of potent SHP2 inhibitors, SHP2 might thus be a potential target for the treatment of fibrosis.

## Abstract (web summary)

Hyperactivation of TGFβ signaling is a common feature of fibrotic diseases. Here the authors show that genetic or pharmacologic inactivation of the tyrosine phosphatase SHP2 prevents TGFβ-induced JAK2/STAT3 signaling, inhibits fibroblast activation and exerts potent anti-fibrotic effects.

## Introduction

Fibrotic diseases are characterized by an excessive accumulation of extracellular matrix, which destroys the physiological architecture of affected tissues and often leads to severe dysfunction of the involved organs. Fibrotic tissue responses can affect virtually every organ and can manifest either as local or systemic fibrotic disease. Systemic sclerosis (SSc) is a prototypical systemic fibrotic disease that can affect multiple organ systems including the skin, the lungs, the heart and the intestine^1^. Although most individual fibrotic diseases have a low incidence, fibrotic tissue responses in chronic disease are highly prevalent, constituting a major burden on modern societies accounting for up to 45% of deaths in the developed world^2,3^.

Fibroblasts are key effector cells in fibrotic diseases. Upon activation, resting fibroblasts can acquire a myofibroblast phenotype, which is characterized by expression of contractile proteins and enhanced release of extracellular matrix^4^. While myofibroblasts are only temporarily observed during physiological tissue remodeling, they remain stably activated in fibrotic diseases. Transforming growth factor-β (TGFβ) is a core pathway of fibroblast activation in physiologic and pathologic conditions and plays a central role for the persistent activation of fibroblasts in fibrotic diseases^5–7^. TGFβ signaling occurs only temporarily in wound healing, but remains active in fibrotic diseases. Fibroblasts isolated from patients with fibrotic diseases demonstrate a TGFβ-biased gene expression signature. Moreover, prolonged activation of TGFβ signaling in mice by fibroblast-specific overexpression of constitutively active TGFβ receptor type I results in a systemic fibrotic disease, whereas targeted inhibition of TGFβ signaling ameliorates fibrosis^1^. Uncontrolled and prolonged activation of TGFβ signaling is thus sufficient and required to induce persistent fibroblast activation and tissue fibrosis^8^. Although the central role of TGFβ in the pathogenesis of fibrotic diseases is well established, it remains still enigmatic why TGFβ signaling is not appropriately terminated in fibrotic diseases. Identification of central checkpoints and re-establishment of effective feedback regulation of TGFβ signaling might offer potential targeted therapies for fibrotic diseases.

SHP2, encoded by the *PTPN11* gene, is a ubiquitously expressed non-receptor tyrosine phosphatase (PTP). SHP2 contains two N-terminal Src homology 2 (SH2) domains, a catalytic PTP domain and a C-terminal tail with two tyrosyl phosphorylation sites^9^. While SHP2 is normally inactive in its basal state, binding to phosphotyrosyl residues of substrate proteins induces conformational changes that activate its phosphatase activity^10^. SHP2 plays a complex role in the regulation of multiple signaling cascades^11,12^. SHP2 has been shown to modulate signaling pathways activated by growth factors such as platelet-derived growth factor (PDGF), epidermal growth factor (EGF), fibroblast growth factor (FGF) and insulin-like growth factor-1 (IGF-1), by interferons and by cytokines such as interleukin (IL)-3, IL-6, granulocyte-macrophage colony-stimulating factor (GM-CSF), as well as by peptide hormones such as erythropoietin (EPO) and insulin. SHP2 participates in signal transduction of various intracellular pathways including RAS/RAF/mitogen-activated protein kinase (MAPK), Janus kinase/signal transducer and activator of transcription (JAK/STAT) and phosphatidylinositol-3 (PI3) kinase pathways^11–13^. However, SHP2 does not only modulate multiple pathways, but may act at multiple sites within a single signaling pathway to modulate the signal relay. For instance, SHP2 directly interacts with cytokine and growth factor receptors, but also binds to a variety of signaling intermediates such as GRB2, FRS2, JAK2, the p85 subunit of PI3 kinase, IRS1 and GAB proteins to further modulate the signaling outcome^14,15^. This regulation at multiple levels enables SHP2 to generate a wide range of diverse effects in different cellular contexts. In most cases, SHP2-induced dephosphorylation diminishes the signaling intensity. However, SHP2 can also promote signaling, either by dephosphorylation of endogenous inhibitors at activating sites or by dephosphorylation of inhibitory tyrosine phosphorylation sites^16,17^. Finally, SHP2 may not only modulate signaling by dephosphorylation of target proteins, but also in a phosphatase-independent manner^18^. Altered activity of SHP2 has been implicated in the pathogenesis of multiple diseases. Those include the Noonan syndrome and the Leopard syndrome with inherited mutations of the *PTPN11* gene^9,19^. The activity of SHP2 is also altered in various types of tumors due to acquired mutations of *PTPN11*. In addition, changes in expression and activity of SHP2 have been implicated into the pathogenesis of autoimmune diseases such as systemic lupus erythematosus or rheumatoid arthritis^20–22^.

In our study, we aimed to characterize the role of SHP2 in SSc. We characterize SHP2 as a molecular checkpoint of TGFβ signaling. SHP2 is required for the activation of JAK2 and STAT3 by TGFβ. Inactivation of SHP2 prevents TGFβ-induced JAK2/STAT3 signaling, reduces fibroblast activation and ameliorates experimental fibrosis. These findings might have translational implications as potent inhibitors of SHP2 currently undergo clinical evaluation in cancer.

## Results

### TGFβ induces SHP2 activity

**Fig. 1:**
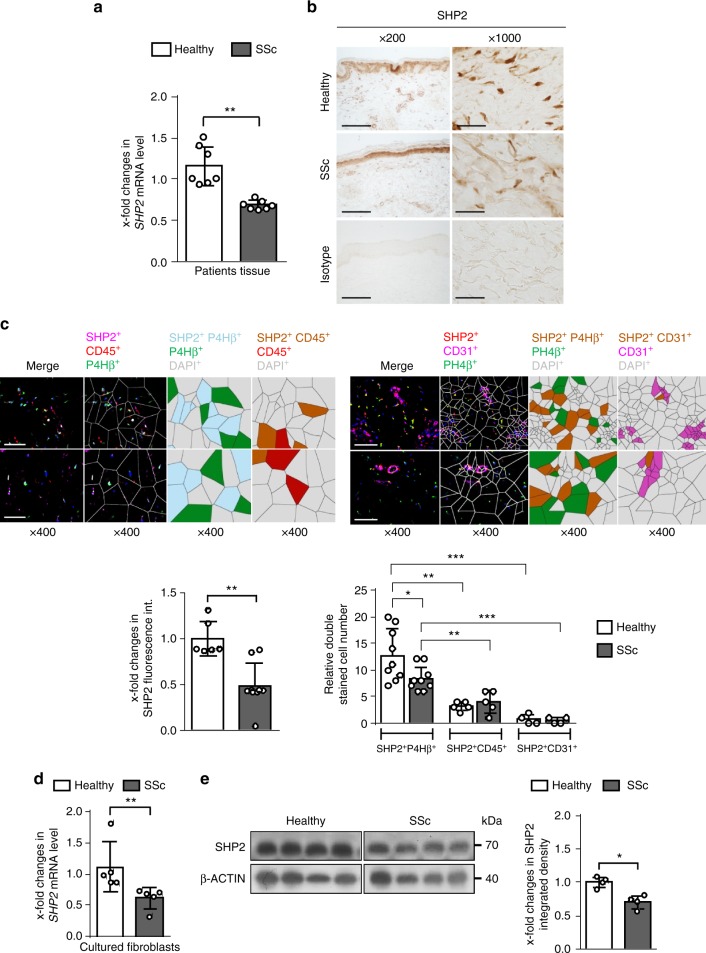
Decreased expression of SHP2 in SSc. **a** The mRNA levels of *SHP2* are significantly reduced in SSc skin as compared to healthy skin (*n* = 7). **b** Immunohistochemistry of SHP2 in SSc skin and matched healthy controls. Representative images are shown at 200- and 1000-fold magnification. **c** Immunofluorescence staining of SHP2 with co-staining for the fibroblast marker P4Hβ, the endothelial cell marker CD31 and the leukocyte marker CD45, and DAPI. SSc fibroblasts demonstrated a reduced staining for SHP2 compared to healthy control. Representative images are shown at 400-fold magnification. Immunofluorescence pictures were processed to generate Voronoi tessellated pictures amenable to computational simulation. Quantification of SHP2 staining intensity (*n* = 5) and of SHP2-positive cells (*n* = 5). **d**, **e** The mRNA (*n* = 5) (**d**) and protein level (*n* = 4) (**e**) of SHP2 are decreased in cultured SSc fibroblasts. Horizontal scale bar, for all images, 500 μm. All data are presented as median ± s.e.m. The *p* values are expressed as follows: 0.05 > *p* > 0.01*; 0.01 > *p* > 0.001**; *p* < 0.001***. Significance was determined by Mann–Whitney test. SSc: systemic sclerosis, Healthy: healthy individual, int.: intensity

To investigate the role of SHP2 in the pathogenesis of SSc, we first analyzed the expression pattern of SHP2 in skin biopsies of SSc patients and healthy controls. The messenger RNA (mRNA) levels of *SHP2* were modestly but statistically significantly decreased in fibrotic skin of SSc patients compared to matched healthy individuals (Fig. 1a). This downregulation was confirmed by immunohistochemistry (Fig. 1b) and immunofluorescence staining (Fig. 1c). Double staining with the fibroblast marker prolyl-4-hydroxylase-β (P4Hβ), the endothelial marker CD31 and the leukocyte marker CD45 demonstrated that fibroblasts account for most of the SHP2 expression in the dermis and that SSc fibroblasts express reduced levels of SHP2 compared to fibroblasts in healthy skin (Fig. 1c). Quantification of the staining further confirmed the decrease of SHP2 in SSc fibroblasts compared to those in healthy skin (Fig. 1c). The mRNA (Fig. 1d) and protein levels (Fig. 1e) of SHP2 were also decreased in cultured SSc fibroblasts as compared to fibroblasts from healthy individuals. The expression of Shp2 was also modestly downregulated in murine models of SSc. The mRNA and protein levels of Shp2 were decreased by 35–45% in the skin of bleomycin-challenged mice and in TSK1 mice. Co-staining of Shp2 with vimentin demonstrated reduced expression of Shp2 in fibroblasts in fibrotic murine skin (Supplementary Fig. 1a–b).

**Fig. 2:**
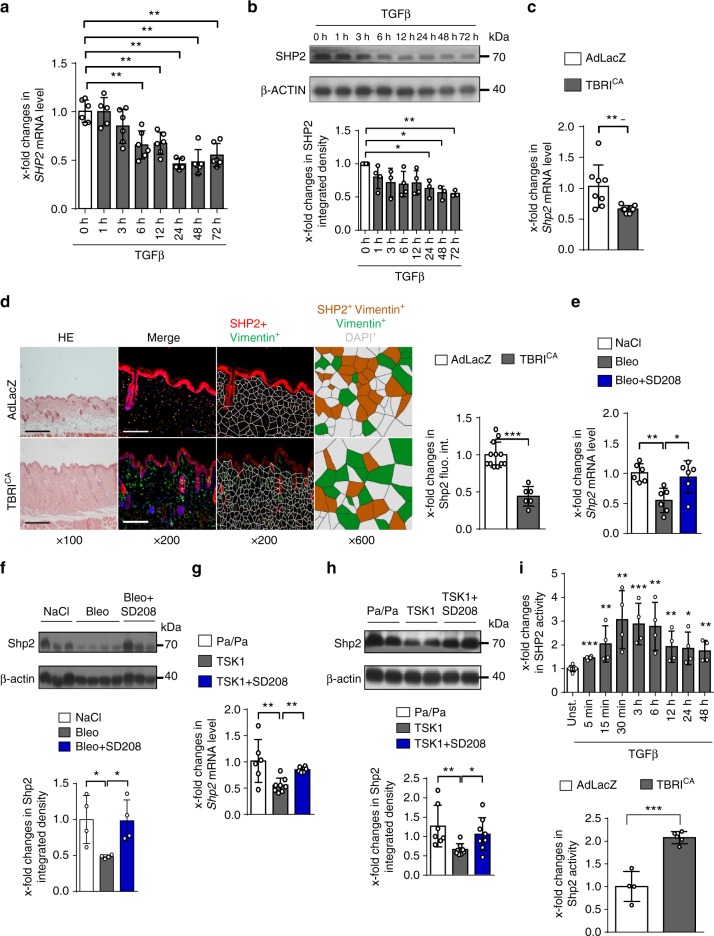
SHP2 is downregulated in TGFβ signaling. **a**, **b** Decreased mRNA (*n* = 6) (**a**) and protein (*n* = 4) (**b**) levels of SHP2 in healthy fibroblasts stimulated with TGFβ (10 ng/ml) for different time points as measured by RT-PCR and western blot, respectively. **c**, **d** Overexpression of TBRI^CA^ (6.67 × 10^7^ IFUs every 2 weeks) significantly reduced mRNA (*n* = 8) and the protein levels of Shp2 in murine skin as shown by qPCR (**c**) and immunofluorescence staining (**d**) of Shp2 with co-staining for fibroblast marker Vimentin and DAPI (*n* ≥ 6 per each group). Representative images are shown at 100–200- and 600-fold magnification. Horizontal scale bar, 500 μm. Immunofluorescence pictures were analyzed by Voronoi tessellation. **e**, **f** Treatment with the selective TGFβ receptor type 1 kinase inhibitor SD208 (60 mg/kg/day) reversed the decrease of *Shp2* mRNA (*n* = 6) (**e**) and protein (*n* = 4) (**f**) in bleomycin-challenged mice (50 µg every other day). **g**, **h** Treatment with the selective TGFβ receptor type 1 kinase inhibitor SD-208 reversed the decrease of *Shp2* mRNA (*n* = 6) (**g**) and protein (**h**) in TSK1 mice (2 mg tamoxifen over 5 days) (*n* ≥ 6 per each group). **i** Phosphatase activity assay. Increases in SHP2 activity after TGFβ stimulation (10 ng/ml) (*n* = 4) in cultured fibroblasts and upon overexpression of TGFβRI (6.67 × 10^7^ IFUs) in murine skin (*n* ≥ 4 per each group). Results shown are representative of three independent experiments. All data are presented as median ± s.e.m. The *p* values are expressed as follows: 0.05 > *p* > 0.01*; 0.01 > *p* > 0.001**; *p* < 0.001***. Significance was determined by Mann–Whitney test. AdLacZ: adenovirus LacZ, TBRI^CA^: constitutively active TGFβ receptor type I, TSK1: Tight skin, Bleo: bleomycin, Pa/Pa: control for TSK1, fluo.: fluorescence, int.: intensity, Unst.: unstimulated

We next investigated the molecular mechanisms underlying the decreased expression of SHP2 in fibrotic tissues. As activation of TGFβ signaling is a common denominator of fibrotic conditions, we analyzed potential effects of TGFβ on SHP2 expression. Indeed, stimulation of human fibroblasts with recombinant TGFβ reduced the mRNA and protein levels of SHP2 with maximal effects after 24 h and 72 h, respectively (Fig. 2a, b). Furthermore, activation of TGFβ signaling by overexpression of a constitutively active TGFβ receptor type I (TBRI^CA^) downregulated *Shp2* mRNA and protein levels in murine skin (Fig. 2c, d). In contrast, inhibition of TGFβ signaling by treatment with the selective TBRI inhibitor SD-208 prevented the downregulation of Shp2 in experimental fibrosis (Fig. 2e–h).

We next investigated the effects of TGFβ on SHP2 activity using phosphatase assays. In contrast to its inhibitory effects on SHP2 expression, TGFβ increased SHP2 activity in human dermal fibroblasts (Fig. 2i). Stimulation of SHP2 activity by TGFβ was observed as early as within 5 min and reached a plateau between 30 min and 6 h. The less pronounced upregulation of SHP2 phosphatase activity by TGFβ at later time points parallels the inhibitory effects of TGFβ on SHP2 expression and may thus result from reduced SHP2 levels. Activation of TGFβ also stimulated Shp2 activity in vivo as demonstrated by increased Shp2 activity in the skin of mice overexpressing TBRI^CA^ as compared to control mice (Fig. 2i).

### Shp2 regulates TGFβ-induced fibroblast activation

**Fig. 3:**
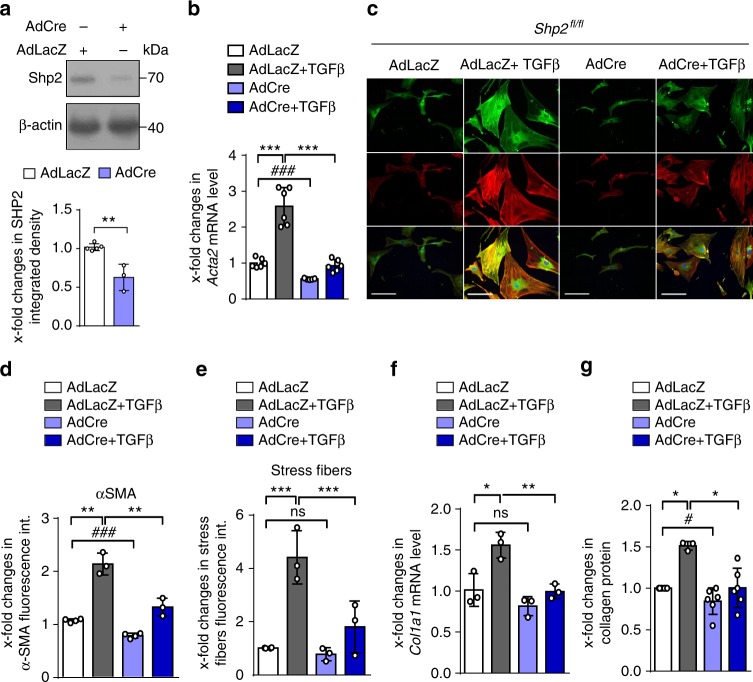
Shp2 regulates TGFβ induced fibroblast activation. **a** Western blot for efficiency of Cre-mediated (80 IFUs/cell) knockout of *Shp2* in murine *Shp2*^*fl/fl*^ fibroblasts (*n* ≥ 3 per each group). **b** *Shp2* knockout decreased mRNA levels of *Acta2* (*n* = 6). **c**–**e** *Shp2* knockout decreased α-SMA and stress fiber staining. Representative images are shown at 200-fold magnification (**c**). Horizontal scale bar, 500 μm. Quantification of α-SMA staining intensity (**d**) and stress fiber staining intensity (**e**) (*n* ≥ 3 different lines). **f**, **g** *Col1a1* mRNA (**f**) and collagen protein release (**g**) induced by TGFβ (10 ng/ml for 24 h) (*n* ≥ 3 different lines). Results shown are representative of three independent experiments. All data are presented as median ± s.e.m. The *p* values are expressed as follows: 0.05 > *p* > 0.01* or ^#^; 0.01 > *p* > 0.001**; *p* < 0.001*** or ^###^; ns: not significant. Significance was determined by Mann–Whitney test. AdCre: adenovirus Cre, AdLacZ: adenovirus LacZ, int.: intensity

To investigate the functional effects of decreased Shp2 levels, we knocked out *Shp2* by infecting dermal fibroblasts from *Shp2*^*fl/fl*^ mice with AdCre (Fig. 3a). Knockout of *Shp2* ameliorated TGFβ-induced myofibroblast differentiation with reduced mRNA and protein levels of α-smooth muscle actin (α-SMA) and impaired formation of stress fibers (Fig. 3b–e). The induction of *Col1a1* mRNA and of collagen protein by TGFβ was also reduced in *Shp2*-deficient fibroblasts (Fig. 3f, g).

### Knockout of *SHP2* ameliorates fibrosis

To generate mice with fibroblast-specific, tamoxifen-inducible deletion of *Shp2*, we crossbred mice harboring *Shp2* alleles flanked by loxP sites (*Shp2*^*fl/fl*^) with mice expressing *Cre*ER under the control of a tamoxifen-inducible, fibroblast-specific type I collagen promoter (Col1a2-CreER)^8^. In the absence of fibrotic stimuli, mice with fibroblast-specific knockout of *Shp2* (referred to as *Shp2* Fib KO) did not show an overt phenotype and the skin architecture and the collagen content were comparable to *Shp2*^*fl/fl*^x*Col1a2-CreER* mice injected with corn oil (referred to as control mice).

**Fig. 4:**
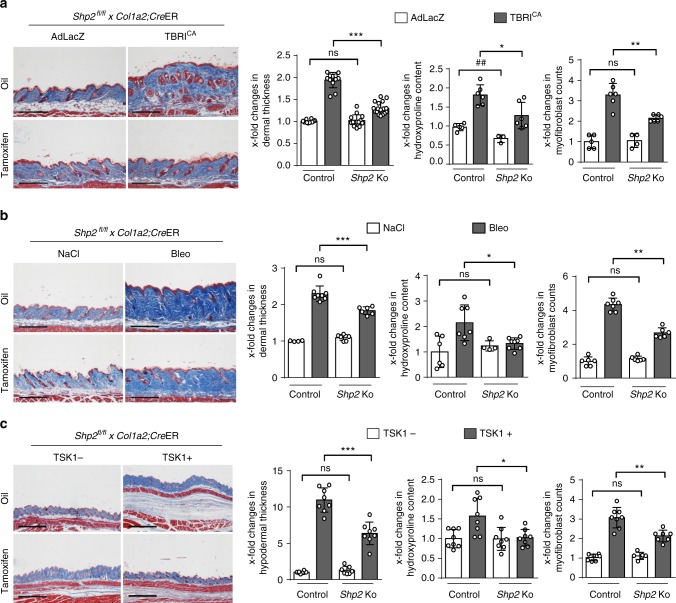
Fibroblast-specific knockout of *Shp2* protects from experimental fibrosis. **a** TBRI^CA^-induced fibrosis (6.67 × 10^7^ IFUs every 2 weeks). Representative images of Masson trichrome-stained skin shown at 100-fold magnification. Dermal thickness, hydroxyproline content and myofibroblast counts. All groups consisted of ≥9 mice each. **b** Bleomycin-induced skin (50 µg every other day) fibrosis. Representative images of Masson trichrome-stained skin shown at 100-fold magnification. Dermal thickness, hydroxyproline content and myofibroblast counts. All groups consisted of ≥8 mice each. **c** TSK1 model (2 mg tamoxifen over 5 days). Representative images of Masson trichrome-stained skin shown at 40-fold magnification. Hypodermal thickness, hydroxyproline content and myofibroblast counts. All groups consisted of ≥8 mice each. Horizontal scale bar in all images, 500 μm. All data are presented as median ± s.e.m. The *p* values are expressed as follows: 0.05 > *p* > 0.01*; 0.01 > *p* > 0.001** or ^##^; *p* < 0.001***; ns: not significant. AdLacZ: adenovirus LacZ, TBRI^CA^: constitutively active TGFβ receptor type I, TSK1: Tight skin, Bleo: bleomycin, Shp2 Ko: SHP2 fibroblast-specific knockout

However, *Shp2* Fib KO mice were protected from experimental skin fibrosis in different mouse models. Fibroblast-specific knockout of *Shp2* ameliorated TBRI^CA^-induced fibrosis with reduced dermal thickening, decreased myofibroblast counts and lower hydroxyproline content upon overexpression of TBRI^CA^ as compared to control mice (Fig. 4a). *Shp2* Fib KO mice were also protected from bleomycin-induced skin fibrosis as an inflammation-driven model of fibrosis with decreased dermal thickening, impaired myofibroblast differentiation and reduced hydroxyproline content as compared to control littermates (Fig. 4b).

TSK1 mice represent a genetic model of fibrosis with endogenous, TGFβ-dependent activation of fibroblasts. Fibroblast-specific knockout of *Shp2* reduced the hypodermal thickening, the hydroxyproline content and the myofibroblast counts in the TSK1 model (Fig. 4c).

### Knockout of * Shp2* inhibits JAK2/STAT3 signaling

**Fig. 5:**
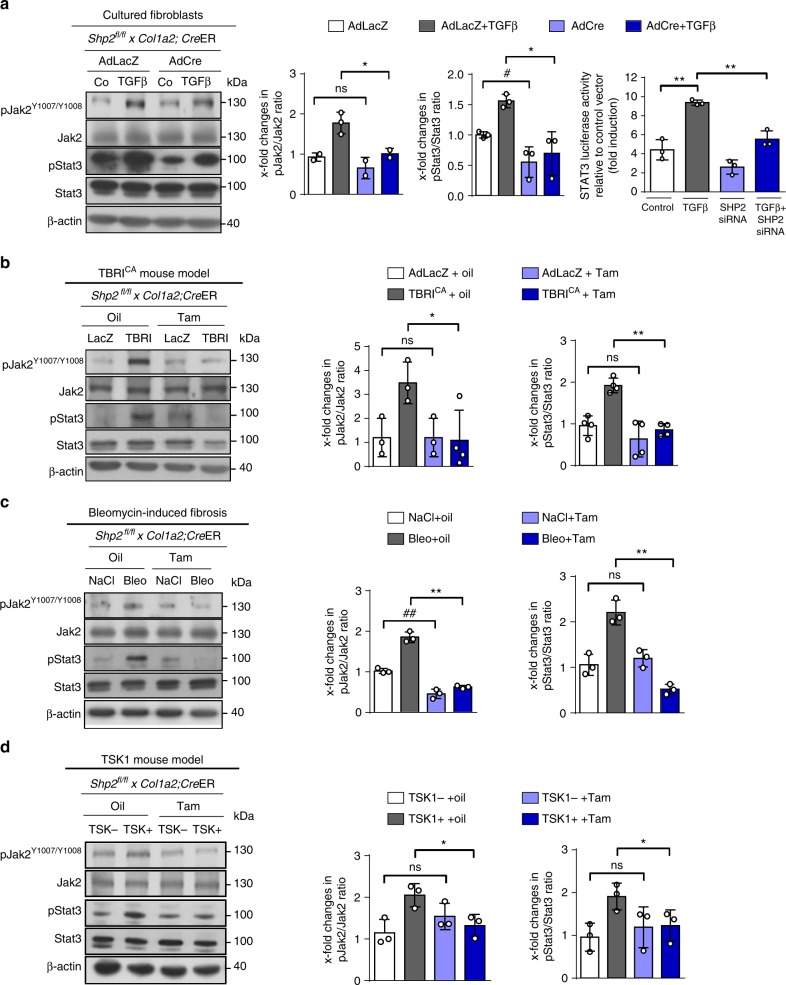
Knockout of *Shp2* decreases JAK2/STAT3 signaling. **a** Knockout of *Shp2* in fibroblasts (*Shp2*^*fl/fl*^ *x* *Col1a2*;*Cre*ER) decreases the levels of pJAK2 and pSTAT3 and STAT3 reporter activity in cultured fibroblasts (*n* = 3 different lines). Cells were stimulated with TGFβ (10 ng/ml for 6 h). **b**–**d** Conditional knockout of *Shp2* reduces the levels of pJAK2 and pSTAT3 in TBRI^CA^ (6.67 × 10^7^ IFUs every 2 weeks) (**b**) and bleomycin-induced fibrosis (50 µg every other day) (**c**) and in TSK1 mice (2 mg tamoxifen over 5 days) (**d**) (*n* ≥ 3). Results shown are representative of three independent experiments. All data are presented as median ± s.e.m. The *p* values are expressed as follows: 0.05 > *p* > 0.01* or ^#^; 0.01 > *p* > 0.001** or^##^; *p* < 0.001***; ns: not significant. Significance was determined by Mann–Whitney test. AdLacZ or LacZ: adenovirus LacZ, TBRI^CA^ or TBRI: constitutively active TGFβ receptor type I, TSK1: Tight skin, Bleo: bleomycin, Tam: tamoxifen, Co: Control unstimulated, AdCre: adenovirus Cre

SHP2 has been shown to regulate angiotensin-II-induced activation of JAK/STAT signaling in vascular smooth muscle cells^4^, mesangial cells^23^ and hepatocellular carcinoma cells^24^. The effects of SHP2 on JAK/STAT have been shown to be highly context and/or cell type dependent: while SHP2 inhibits JAK/STAT signaling in leukocytes under inflammatory conditions, e.g., upon stimulation with IL-6^13,25^, it promotes JAK/STAT signaling in mesenchymal cells under non-inflammatory conditions, e.g., upon stimulation with angiotensin^26,27^. JAK2 and STAT3 have recently been identified as downstream mediators of TGFβ signaling in fibrosis^28–30^. We therefore aimed to investigate whether SHP2 may modulate TGFβ signaling by regulation of JAK2/STAT3 signaling. JAK2 activation was assessed by three approaches. First, by analysis of the phosphorylation status of JAK2 at Y1007/Y1008 (pJAK2^Y1007/Y1008^), as phosphorylation of JAK2 at this particular site is considered as a key step in the activation of JAK2. As a second readout, we analyzed phosphorylation of STAT3 at Y705 (pSTAT3^Y705^), as STAT3 is a main downstream target of JAK2. Finally, we quantified changes in STAT3-dependent transcription in reporter studies. Knockout of *Shp2* in cultured fibroblasts inhibited TGFβ-induced JAK2/STAT3 signaling with reduced levels of pJAK2^Y1007/Y1008^ and decreased levels of pSTAT3 (Fig. 5a). The total expression levels of JAK2 and STAT3 did not change. Knockdown of *SHP2* also ameliorated STAT3-dependent reporter activity (Fig. 5a). The impaired activation of JAK2/STAT3 signaling upon inactivation of *Shp2* was confirmed in experimental fibrosis. Fibroblast-specific knockout of *Shp2* was associated with decreased levels of pJAK2^Y1007/Y1008^ and pSTAT3^Y705^ in TBRI^CA^- (Fig. 5b) and bleomycin-induced fibrosis (Fig. 5c) as well as in TSK1 mice (Fig. 5d) compared to corresponding controls.

### SHP2 regulates TGFβ signaling via its phosphatase activity

**Fig. 6:**
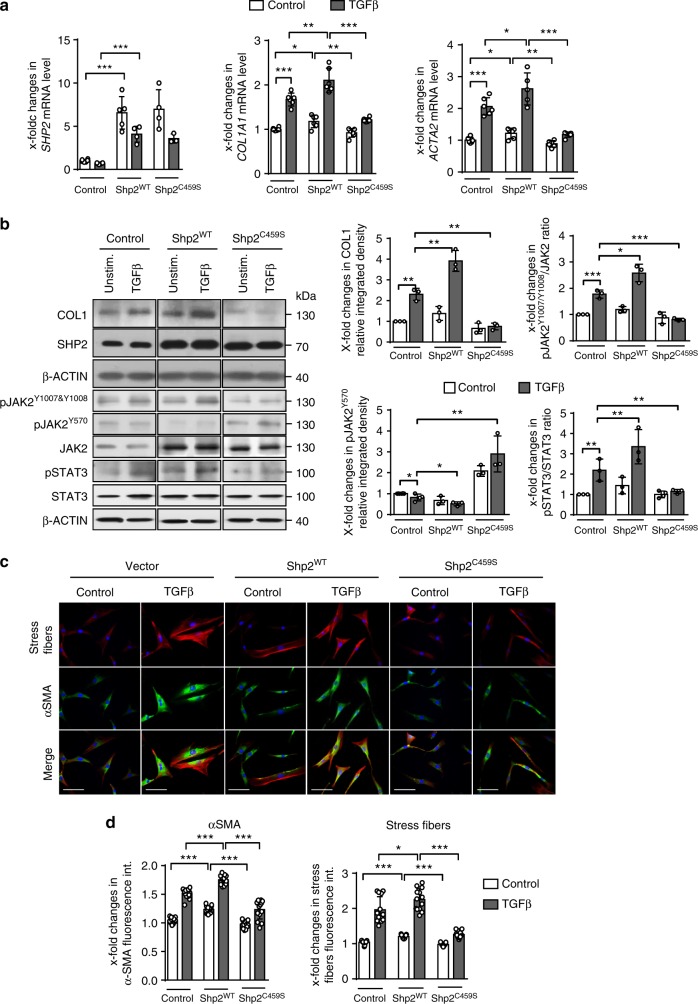
SHP2 enhances TGFβ-induced fibroblast activation via JAK2/STAT3. **a** mRNA levels of *SHP2* after overexpression in human dermal fibroblasts. mRNA levels of *COL1A1* in human fibroblasts transfected with empty vector, SHP2^WT^- and SHP2^C459S^-expression vectors, with or without TGFβ1 treatment (10 ng/ml for 24 h) (*n* ≥ 4). **b** Western blot analysis and respective quantifications for type I collagen and SHP2 in human fibroblasts transfected with empty vector, SHP2^WT^- and SHP2^C459S^-expression vectors, with or without TGFβ1 treatment (10 ng/ml for 24 h). Western blot for pJAK2^Y1007/Y1008^, pJAK2^Y570^, total JAK2, pSTAT3^Y705^ and total STAT3 with β-actin as loading control (TGFβ 10 ng/ml for 6 h) (*n* = 3). Results shown are representative of three independent experiments. **c**, **d** Representative images of immunofluorescence stainings for α-SMA and stress fiber staining are shown at 400-fold magnification (**c**) and quantification of α-SMA staining intensity as well as stress fiber staining intensity (**d**) (*n* ≥ 4). Horizontal scale bar, 500 μm. All data are presented as median ± s.e.m. The *p* values are expressed as follows: 0.05 > *p* > 0.01*; 0.01 > *p* > 0.001**; *p* < 0.001***. Significance was determined by Mann–Whitney test. Vector: empty vector, SHP2^WT^: plasmid carrying full length of *SHP2* wild-type gene, SHP2^C459S^: plasmid carrying a phosphatase-dead mutant of *SHP2*, unstim.: unstimulated, int.: intensity

SHP2 can regulate growth factor signaling by dephosphorylation of target proteins or in a phosphatase-independent manner^18^. To investigate whether the phosphatase activity of SHP2 is required for regulation of TGFβ signaling in fibroblasts, we overexpressed a phosphatase-dead mutant of SHP2 (SHP2^C459S^) in cultured human fibroblasts and compared the effects of overexpression of SHP2^C459S^ and the non-mutated SHP2 (SHP2^WT^) on TGFβ signaling and fibroblast activation. Overexpression of SHP2^WT^ increased the mRNA levels of *COL1A1* and *ACTA2*, increased the release of collagen (Fig. 6a, b) and upregulated the levels of α-SMA and the formation of stress fibers (Fig. 6c, d) as compared to control cells transfected with the empty coding vector. In contrast, overexpression of SHP2^C459S^ did not enhance fibroblast activation, but rather acted in a dominant negative manner to suppress TGFβ-induced fibroblast activation (Fig. 6c, d). Consistently, JAK2/STAT3 signaling was enhanced by overexpression of SHP2^WT^ with decreased levels of pJAK2^Y570^ and increased levels of pJAK2^Y1007/Y1008^ and pSTAT3^Y705^, while it was found suppressed by overexpression of SHP2^C459S^ (Fig. 6b).

To further confirm that SHP2 regulates TGFβ-induced JAK2/STAT3 signaling by dephosphorylation of JAK2 at the inhibitory phosphorylation site at Y570, we overexpressed a mutant JAK2 resistant to phosphorylation at Y570 (JAK2^ΔY570F^) in fibroblasts.

**Fig. 7:**
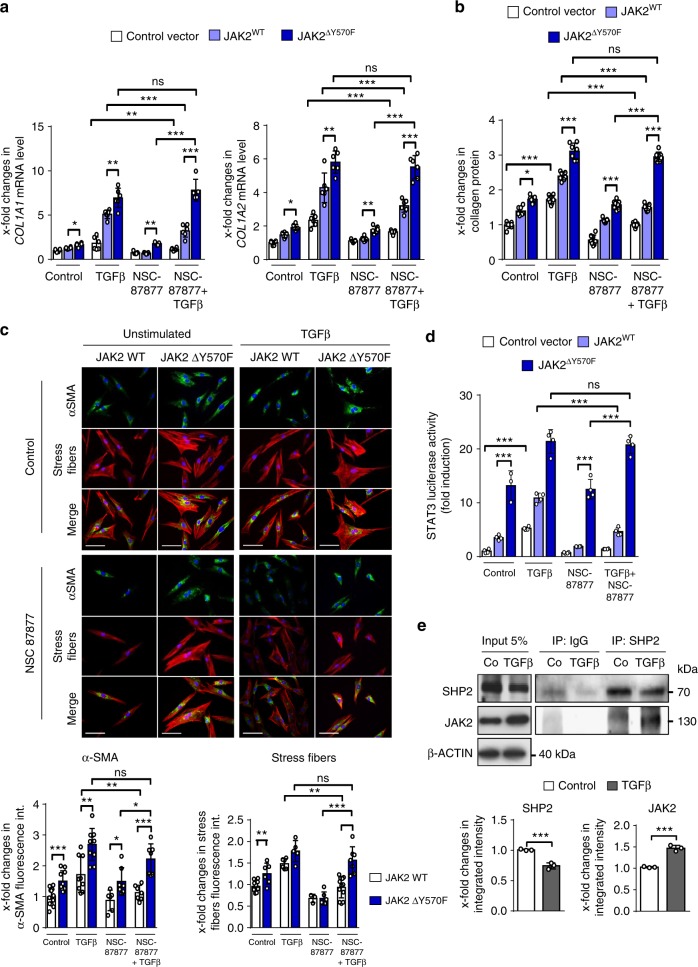
Overexpression of JAK2^∆Y570F^ prevents the inhibitory effects of SHP2 inhibitors on TGFβ-induced fibroblast activation. **a** mRNA levels of *COL1A1* and *COL1A2* (TGFβ 10 ng/ml for 24 h) (*n* ≥ 5). **b** Release of collagen protein (TGFβ 10 ng/ml for 24 h) (*n* ≥ 6). **c** Representative images of immunofluorescence stainings for α-SMA and stress fiber at 400-fold magnification and respective quantifications (TGFβ 10 ng/ml for 24 h) (*n* ≥ 6). Horizontal scale bar, 500 μm. **d** STAT3 reporter Assay upon JAK2 WT and Y570F mutant overexpression. Cells were treated with TGFβ (10 ng/ml for 6 h) and NSC-87877 (100 µM) (*n* ≥ 4). **e** Co-immunoprecipitation and respective quantifications of endogenous JAK2 with endogenous SHP2 in human fibroblasts stimulated with TGFβ (10 ng/ml for 30′) (*n* = 3). Results shown are representative of ≥ 3 independent experiments. All data are presented as median ± s.e.m. The *p* values are expressed as follows: 0.05 > *p* > 0.01*; 0.01 > *p* > 0.001**; *p* < 0.001***; ns: not significant. Significance was determined by Mann–Whitney test. JAK2^WT^: JAK2 Wild type, JAK2^ΔY570F^: JAK2 mutant resistant to phosphorylation at Y570, NSC-87877: SHP1/SHP2 inhibitor, Co: control unstimulated, int.: intensity, IP: immunprecipitation

Overexpression of JAK2^ΔY570F^ promoted activation of resting fibroblasts and rendered them more susceptible to the stimulatory effects of TGFβ as compared to fibroblasts transfected with control vector or non-mutated JAK2 vectors (Fig. 7a–c). Treatment with NSC-87877, an inhibitor of both SHP1 and SHP2, ameliorated the stimulatory effects of TGFβ in fibroblasts transfected with control vector or with non-mutated JAK2 constructs. However, fibroblasts overexpressing JAK2^ΔY570F^ were insensitive to SHP2 inhibition. Treatment with NSC-87877 did not decrease the mRNA levels of *COL1A1* and *COL1A2*, the release of collagen protein, α-SMA expression and stress fiber formation in fibroblasts overexpressing JAK2^ΔY570F^ (Fig. 7a–c). Similar findings were obtained in STAT3 reporter assays (Fig. 7d). Overexpression of JAK2^ΔY570F^ increased STAT3 reporter activity and cells overexpressing JAK2^ΔY570F^ were insensitive to the inhibitory effects of SHP2 inhibition on STAT3 reporter activity. We next aimed to show that TGFβ promotes binding of SHP2 to JAK2. Indeed, stimulation of fibroblasts with TGFβ promoted interaction of SHP2 with JAK2 and increased amounts of JAK2 precipitated with SHP2 in fibroblasts upon stimulation with TGFβ (Fig. 7e). Together, these data demonstrate that TGFβ induces SHP2-dependent dephosphorylation of JAK2 at Y570 to promote activation of STAT3.

As NSC-87877 does not discriminate between SHP1 and SHP2 and may also inhibit other tyrosine phosphatases in higher concentrations (half-maximal inhibitory concentration (IC_50_) for SHP2 is 0.318 µM, for SHP1 0.355 µM, for PTP1b = 1.691 µM and for HePTP = 7.745 µM), we aimed to confirm our findings with more specific SHP2 inhibitors. To better discriminate between SHP1- and SHP2-mediated effects, we confirmed our results with SHP099 (allosteric inhibitor) and 11-a1 (active site inhibitor), both of which possess high selectivity for SHP2 over SHP1^20,31,32^. Both inhibitors effectively reduced TGFβ-induced fibroblast activation to an extent similar to that observed with NSC-87877 (Supplementary Fig. 2a–c).

### Inhibition of *SHP2* exerts anti-fibrotic effects

**Fig. 8:**
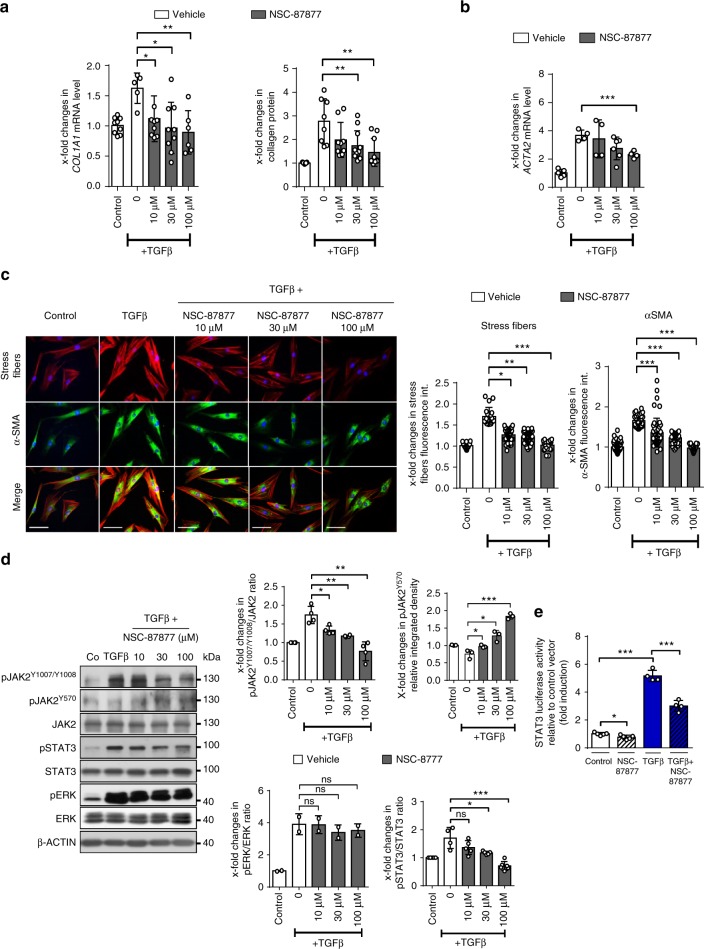
Inhibition of SHP2 limits JAK2/STAT3 signaling and fibroblast activation. **a** Changes in the mRNA levels of *COL1A1 and* of collagen protein in human fibroblasts incubated with increasing doses of NSC-87877 (10 µM, 30 µM and 100 µM). Fibroblasts were treated with TGFβ (10 ng/ml) for 24 h. **b** *ACTA2* mRNA. (*n* ≥ 4) **c** Representative images of immunofluorescence stainings for α-SMA and stress fiber staining are shown at 400-fold magnification and quantification of α-SMA staining intensity as well as stress fiber staining intensity (*n* ≥ 15) (TGFβ 10 ng/ml for 24 h). Horizontal scale bar, 500 μm. **d** Representative western blots for pJAK2^Y1007/Y1008^, pJAK2^Y570^, total JAK2, pSTAT3^Y705^ and total STAT3 with β-actin as loading control and quantification of the results (TGFβ 10 ng/ml for 6 h) (*n* ≥ 2). **e** Changes in STAT3 reporter activity (*n* ≥ 6) (TGFβ 10 ng/ml for 6 h). Results shown are representative of three independent experiments All data are presented as median ± s.e.m. The *p* values are expressed as follows: 0.05 > *p* > 0.01*; 0.01 > *p* > 0.001**; *p* < 0.001***; ns: not significant. Significance was determined by Mann–Whitney test. NSC-87877: SHP1/SHP2 inhibitor, Co: control unstimulated, int.: intensity

After demonstrating that fibroblast-specific genetic inactivation of *Shp2* ameliorates experimental fibrosis, we next aimed to investigate the anti-fibrotic potential of pharmacological inhibition of SHP2. Incubation with NSC-87877 ameliorated the stimulatory effects of TGFβ on *COL1A1* mRNA and release of collagen protein (Fig. 8a) and inhibited myofibroblast differentiation with reduced mRNA (Fig. 8b) and protein levels of α-SMA and impaired formation of stress fibers (Fig. 7c) at non-toxic concentrations (Supplementary Fig. 3a). In accordance with our proposed mode of action (Supplementary Fig. 4a-b), incubation with NSC-87877 inhibited the accumulation of pJAK2^Y1007/Y1008^ and of its downstream target pSTAT3, but increased the levels of pJAK2^Y570^ in TGFβ-stimulated fibroblasts (Fig. 8d). Consistently, incubation with NSC-87877 inhibited the TGFβ-induced activation of STAT3-dependent transcription in reporter assays (Fig. 8e).

**Fig. 9:**
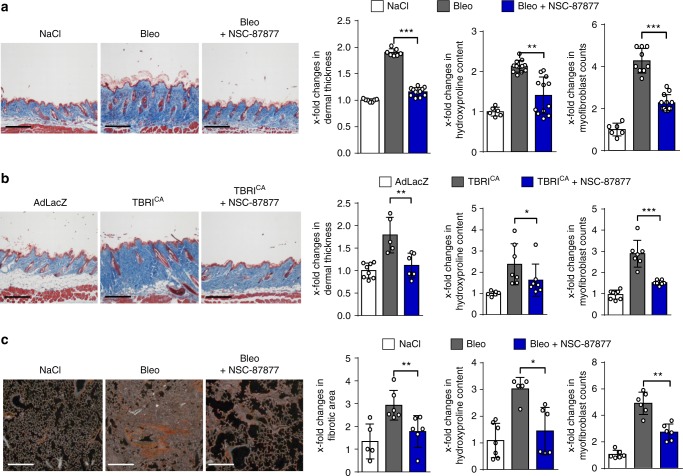
Treatment with NSC-87877 ameliorates experimental fibrosis. The SHP1/SHP2 inhibitor NSC-87877 was applied at doses of 5 mg/kg q.d. **a** Bleomycin-induced skin (50 µg every other day) fibrosis: representative images of Masson trichrome-stained skin shown at 100-fold magnification. Dermal thickness, hydroxyproline content and myofibroblast counts. **b** TBRI^CA^-induced (6.67 × 10^7^ IFUs every 2 weeks) skin fibrosis: representative images of Masson trichrome-stained skin shown at 100-fold magnification. Dermal thickness, hydroxyproline content and myofibroblast counts. **c** Bleomycin-induced lung fibrosis (50 µg single doses): representative images of Sirius red-stained lung shown at 100-fold magnification. Quantification of Sirius red-positive area (fibrotic area), hydroxyproline content and myofibroblast counts. All groups in all models consisted of ≥5 mice each. Horizontal scale bar in all images, 500 μm. All data are presented as median ± s.e.m. The *p* values are expressed as follows: 0.05 > *p* > 0.01*; 0.01 > *p* > 0.001**; *p* < 0.001***. Significance was determined by Mann–Whitney test; Bleo: bleomycin, TBRI^CA^: constitutively active TGFβ receptor type I, AdLacZ: adenovirus LacZ

Treatment with NSC-87877 also ameliorated bleomycin-induced skin fibrosis with decreased dermal thickening, hydroxyproline content and myofibroblast counts as compared to vehicle-treated mice (Fig. 9a). Similar results were obtained in the TBRI^CA^-induced skin fibrosis (Fig. 9b). In addition, NSC-87877 also ameliorated bleomycin-induced pulmonary fibrosis (Fig. 9c).

**Fig. 10:**
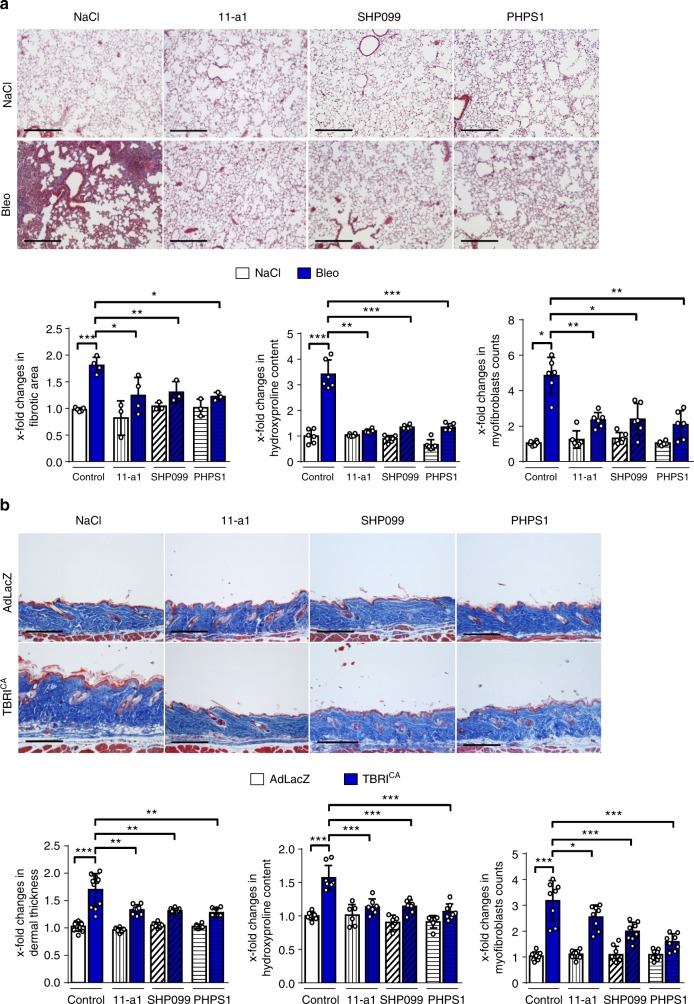
Selective inhibition of Shp2 ameliorates experimental fibrosis. The following doses of SHP2 inhibitors were applied: PHPS1 (5 mg/kg q.d.), SHP099 (75 mg/kg q.d.) and 11-a1 (7.5 mg/kg q.d.). **a** Bleomycin-induced pulmonary fibrosis (50 µg single doses)**:** representative images of Masson trichrome-stained skin shown at 100-fold magnification; fibrotic area, hydroxyproline content and myofibroblast counts. **b** TBRI^CA^-induced dermal fibrosis (6.67 × 10^7^ IFUs every 2 weeks): representative images of Masson trichrome-stained skin shown at 100-fold magnification; Dermal thickness, myofibroblast counts and hydroxyproline content. All groups in both models consisted of ≥4 mice each. Horizontal scale bar for all images, 500 μm All data are presented as median ± s.e.m. The *p* values are expressed as follows: 0.05 > *p* > 0.01*; 0.01 > *p* > 0.001**; *p* < 0.001***. Significance was determined by Mann–Whitney test. Bleo: bleomycin, AdLacZ: adenovirus LacZ, TBRI^CA^: constitutively active TGFβ receptor type I

We employed three different inhibitors of SHP2, the active site inhibitors 11-a1 and PHPS1, as well as the allosteric inhibitor SHP099^31–33^ to confirm the findings obtained with the SHP1/SHP2 inhibitor NSC-87877. This may be of particular importance as SHP1 has been shown to inhibit proliferation of pro-fibrotic hepatic stellate cells and may thus also modulate the outcome of fibrotic diseases^34,35^. 11-a1, PHPS1 and SHP099 all ameliorated bleomycin-induced pulmonary fibrosis (Fig. 10a) and TBRI^CA^-induced dermal fibrosis (Fig. 10b). Hydroxyproline content, myofibroblast counts and fibrotic area or dermal thickness, respectively, were significantly reduced in mice treated with 11-a1, PHPS1 or SHP099 compared to vehicle-treated controls and the anti-fibrotic effects were in the range of those observed with NSC-87877 in previous experiments.

## Discussion

Our data characterize SHP2 as an important regulator of TGFβ signaling in fibroblasts. *Shp2*-deficient murine fibroblasts are less responsive to TGFβ. An impaired response to the pro-fibrotic effects of TGFβ was also observed in human fibroblasts upon pharmacological inhibition of SHP2. Consistent with these findings, inhibition of SHP2 prevented epithelial-to-mesenchymal transition in A549 adenocarcinoma cells^36^. In contrast, ectopic expression of full-length but not of phosphatase-deficient *SHP2* in fibroblasts enhances TGFβ signaling. Mechanistically, SHP2 regulates the TGFβ-dependent activation of JAK2. The tyrosine kinase JAK2 has recently been identified as a pro-fibrotic mediator. Inhibition of JAK2 exerts anti-fibrotic effects and has been shown to ameliorate skin, liver, pulmonary and renal fibrosis^29,37,38^. The activity of JAK2 is tightly regulated by phosphorylation. Depending on the actual target site, these phosphorylation events can either activate or inhibit JAK2. Phosphorylation of JAK2 at Y570 inhibits JAK2 activation. Dephosphorylation of JAK2 at Y570 is a pre-requisite for JAK2 to undergo the activating phosphorylation at Y1007/Y1008, whereas mutation of the Y570 leads to enhanced and prolonged JAK2 activation^39^. We demonstrate that SHP2 can dephosphorylate JAK2 at Y570 to promote TGFβ-dependent activation of JAK2 and its downstream mediator STAT3 (Supplementary Fig. 4a). In contrast, pharmacological inhibition of SHP2, overexpression of a catalytically inactive SHP2 mutant or knockout of *Shp2* inhibits dephosphorylation of pJAK2^Y570^, which prevents phosphorylation of JAK2 at Y1007/Y1008 and subsequent activation of STAT3 in TGFβ-stimulated fibroblasts and in experimental fibrosis^30,40^.

We previously characterized the serine/threonine kinase CK2 as an upstream activator of JAK2/STAT3 signaling in fibroblasts. Pharmacological inhibition of CK2 ameliorated experimental fibrosis and those anti-fibrotic effects were associated with decreased levels of pJAK2^Y1007/1008^ and pSTAT3^40^. However, SHP2 regulates JAK2 signaling at a different level and by a different mechanism than CK2. Although the precise mechanisms by which the serine/threonine kinase CK2 promotes accumulation of pJAK2^Y1007/1008^ and pSTAT3 have not been uncovered, the effects seem to be indirect, given the delayed effects of CK2 on JAK2 signaling. CK2 may thus serve to amplify JAK2 signaling by enhancing positive signaling input. In contrast, SHP2 provides a permissive signaling environment, as it removes the inhibitory phosphorylation of JAK2 at Y570, which is required for subsequent phosphorylation at Y1007/1008 and thus for activation of JAK2 signaling. SHP2-induced removal of this inhibitory phosphorylation mark may thus be a pre-requisite for effective activation of JAK2 signaling, which may explain the potent anti-fibrotic effects of targeting SHP2.

The activating effects of SHP2 on TGFβ-induced JAK2/STAT3 activation directly translate into stimulatory effects on fibroblasts. Fibroblasts overexpressing SHP2 are more susceptible to the pro-fibrotic effects of TGFβ, whereas the stimulatory effects of TGFβ on myofibroblast differentiation and collagen release are reduced in *Shp2* knockout fibroblasts. Selective inactivation of *Shp2* in fibroblasts also reduced the pro-fibrotic effects of TGFβ signaling in vivo. *Shp2* Fib KO mice were protected from experimental fibrosis induced by overexpression of a constitutively active TGFβ receptor type I. Moreover, fibroblast-specific inactivation of *Shp2* also protected from bleomycin-induced skin fibrosis and ameliorated fibrosis in TSK1 mice, thereby confirming the central regulatory function of Shp2 on TGFβ signaling and fibroblast activation in multiple complementary models of SSc. Consistent with the role of SHP2 as a mediator of tissue remodeling, inactivation of *Shp2* in airway epithelial cell reduced pulmonary remodeling in response to ovalbumin challenge as a model of chronic asthma^41^. Our findings are also in line with recent results which show that SHP2 is required for epithelial-to-mesenchymal transition induced by IL-6 in breast cancer cells^42^. Our findings may also be supported by reports about fibrotic changes in patients with Noonan syndrome with hyperactive SHP2 such as myocardial fibrosis^43,44^, fibrosis of the extraocular muscles^45^ and recurrent keloid formation^34^.

The inhibitory effects of SHP2 on fibroblast activation may not be limited to myofibroblasts. Inhibition of SHP2 has recently been shown to ameliorate the responsiveness of synovial fibroblast-like cells from patients with rheumatoid arthritis that exhibit a characteristic inflammatory and invasive phenotype to tumor necrosis factor and PDGF^21,22^. Together, these findings identify SHP2 as a key regulator of growth factor-induced fibroblast activation.

These findings may have translational implications. The critical role of SHP2 in various types of cancer prompted the development of small inhibitors of SHP2^31^, some of which already showed promising results in first clinical trials^46^. The SHP inhibitor NSC-87877, which serves as a lead compound for the development of new SHP inhibitors, exerted anti-fibrotic effects in bleomycin- and TBRI^CA^-induced skin fibrosis in well-tolerated doses. These murine models resemble different stages and subgroups of SSc. The mouse model of bleomycin-induced dermal fibrosis mimics inflammatory stages of SSc, in which fibroblasts are pre-dominantly activated by pro-fibrotic mediators released from infiltrating leukocytes. In contrast, the mouse model of TBRI^CA^-induced fibrosis resembles SSc patients, in which inflammatory infiltrates have largely resolved and fibroblasts are endogenously activated^47^. Moreover, treatment with NSC-87877 also ameliorated pre-established bleomycin-induced pulmonary fibrosis as the leading cause of fibrosis-associated death in SSc. Extrapolating the findings from the mouse models to humans, these findings may indicate that SHP2 plays a crucial role in the pathogenesis of inflammatory as well as non-inflammatory types of fibrotic diseases, that the pro-fibrotic effects of SHP2 are not limited to the skin, but are also operative in the lung and that inhibition of SHP2 is not only effective in preventive, but also in therapeutic settings. However, considering the complex pathogenesis and the heterogeneity of SSc, further in vivo studies are required to confirm these findings. Particular attention should be appointed to the effects of SHP2 inhibition on macrophage polarization. A recent report demonstrated that SHP2 is required for M1 polarization of macrophages in the context of Haemophilus influenza infection^48^ and that inactivation of SHP2 may promote M2 polarization^49^. Macrophage polarization in SSc is skewed towards M2 polarization and those alternatively activated fibroblasts are thought to play an important role in fibroblast activation by the release of pro-fibrotic mediators such as IL-4 and IL-13^50,51^. Indeed, selective knockout of SHP2 may actually promote experimental fibrosis^49^. Thus, a careful selection for patients with less inflammatory activity may be required to ensure that the beneficial effect of SHP2 inhibition on fibroblasts are not outweighed by the effects on M2 polarization^52^.

We focused on the role of SHP2 in the pathogenesis of fibrosis. However, SHP2 is also differentially expressed in microvascular endothelial cells of SSc patients as demonstrated by immunohistochemistry in our study and first evidence links SHP2 to the pathogenesis of vascular diseases. SHP2 has been reported to enhance PDGF signaling during vascular neointima formation^53^ and to be required for angiotensin-II-induced apoptosis of pulmonary endothelial cells^54^. The role of SHP2 in the vascular pathogenesis of SSc requires further investigation in murine models that resemble the vascular alterations in SSc such as Fra-2 transgenic mice or uPAR (urokinase-type plasminogen activator receptor) knockout mice^55,56^.

In summary, we characterize SHP2 as a positive regulator of TGFβ-dependent activation of JAK2/STAT3 signaling. Genetic or pharmacologic inactivation of SHP2 inhibits JAK2/STAT3 signaling, reduces fibroblast activation and ameliorates experimental fibrosis in several complementary models. These findings identify SHP2 as a potential molecular target for the treatment of fibrosis in fibrotic diseases such as SSc.

## Methods

### Patients

Dermal fibroblasts were isolated from skin biopsies of 24 SSc patients and 28 age- and sex-matched healthy volunteers. All patients fulfilled the ACR/EULAR (American College of Rheumatology/European League Against Rheumatism) 2013 criteria^57^. Sixteen patients were female and seven were male. The median age of SSc patients was 49 years (range: 19–72 years), and their median disease duration was 6 years (range: 1–12 years). The human studies were approved by the Ethical committee of the Medical Faculty of the University of Erlangen-Nuremberg. All patients and controls signed a consent form approved by the local institutional review board. All human studies were performed in compliance with the relevant ethical regulations

### Animal studies

Mice carrying two conditional alleles of *Shp2* (*Shp2*^*fl/fl*^) were crossbred with *Col1a2-Cre*ER mice^8^. Cre-mediated recombination was induced by repeated intraperitoneal injections of 2 mg tamoxifen over 5 days. Control groups were injected with corn oil. The role of Shp2 signaling in fibrosis was investigated in three different mouse models. (i) In the model of bleomycin-induced dermal fibrosis (10 weeks of age, mixed genders), fibrosis was induced by local injections of bleomycin (50 µg every other day) for 4 weeks^58^. Subcutaneous injections of 0.9% NaCl served as control. (ii) In the TBRI^CA^ model, injections of replication-deficient type 5 adenoviruses encoding for a constitutively active TBRI construct^8^ induced localized skin fibrosis (12 weeks of age, mixed genders). Mice injected with type 5 adenoviruses encoding for *LacZ* served as controls. 6.67 × 10^7^ infectious units (IFUs) were injected intracutaneously and analyzed after 8 weeks^59^. (iii) TSK1 mice are a genetic model of skin fibrosis (10 weeks of age, mixed genders) with progressive accumulation of extracellular matrix in the hypodermal layer of the skin^47^. TSK1 mice were analyzed at an age of 10 weeks. The effect of SHP2 inhibitors NSC-87877 (Santa Cruz Technologies), PHPS1 (Calbiochem, Darmstadt, Germany), SHP099 (Chemietek, Indianapolis, USA) and 11-a1 (Professor Zhang, Indiana, USA) on experimental fibrosis was evaluated in three animal models: (i) In the bleomycin-induced dermal fibrosis model, treatment with intraperitoneal injection of 5 mg/kg q.d. NSC-87877 was initiated simultaneously with the first bleomycin injection and the outcome was analyzed after 3 weeks. (ii) In the TBRI^CA^ virus-induced dermal fibrosis model (C57BL/6 background 12 weeks of age, mixed genders), treatment with intraperitoneal injections of 5 mg/kg/day NSC-87877, 7.5 mg/kg q.d. 11-a1, 5 mg/kg q.d. PHPS1 or 75 mg/kg q.d. SHP099 via oral gavage was started simultaneously with the first virus injection and the outcome was analyzed after 8 weeks. (iii) Bleomycin-induced pulmonary fibrosis was induced by a single intra-tracheal application of bleomycin (50 µg) in C57BL/6 mice (14 weeks of age, mixed genders) using a high pressure syringe (Penn-Century, Wyndmoor, PA, USA)^60^. Instillation of equal volumes of 0.9% NaCl served as control. Treatment with Shp2 inhibitors was started simultaneously with the instillation of bleomycin and analysis was performed after 4 weeks. In all mouse models, vehicle-treated mice served as controls. TGF-β-RI kinase inhibitor SD208 was injected intraperitoneal in a dose of 60 mg/kg q.d. All animal experiments were approved by the governments of Mittelfranken or Unterfranken, Germany. All animal experiments were performed in compliance with the relevant ethical regulations.

### Cell culture

Human dermal fibroblasts were isolated from 10 SSc patients and 10 age- and sex-matched healthy volunteers. Mouse fibroblasts were isolated from skin biopsies of Shp2-deficient (*Shp2*^*fl/fl*^*Col1a2-Cre*ER) mice and wild-type littermates. After enzymatic digestion of the skin biopsies with dispase II (Merck KGaA, Darmstadt, Germany), cells were cultured in Dulbecco's modified Eagle's medium/F-12 medium containing 10% heat-inactivated fetal calf serum, 25 mM HEPES, 100 U/ml penicillin, 100 μg/ml streptomycin, 2 mM l-glutamine, 2.5 μg/ml amphoteric-in B (all Gibco–Life Technologies, Darmstadt, Germany) and 0.2 mM ascorbic acid (Sigma-Aldrich, Steinheim, Germany). Fibroblasts from passages 4–8 were used for all experiments. Cell lines were tested for mycoplasma contamination^61^. Gene silencing was achieved by transfection of 3 μg short interfering RNA (siRNA) duplexes using the 4D-Nucleofector (Lonza, Cologne, Germany). The siRNA duplexes: human *SHP2* 5′-GGU ACA UCG ACU UCC UCU A-3′ (forward), 5′-UAG AGG AAG UCG AUG UAC C-3′ (reverse); non-targeting siRNAs (Life Technologies, Darmstadt, Germany) served as controls. Cre-mediated recombination in murine fibroblasts isolated from *Shp2*^*fl/fl*^ mice was induced by infection with type 5 adenoviral vectors encoding for *Cre* recombinase (AdCre - 80 IFUs/fibroblast). Type 5 adenoviral vectors encoding for *LacZ* (AdLacZ) served as controls. In selective experiments, cells were incubated with recombinant TGFβ (10 ng/ml) (PeproTech, Hamburg, Germany).

SHP2 activity was inhibited in fibroblasts using the following inhibitors: NSC-87877 (100 µM), SHP099 (1.4 µM) and 11-a1 (0.2 µM). Cells were pretreated with inhibitors 2 h before addition of TGFβ. JAK2 was inhibited using the JAK2 inhibitor TG101209 (500 nM) (Selleckchem, Houston, USA).

### Quantitative real time-PCR

Gene expression was quantified by SYBR Green real time-PCR using the MX3005P Detection System (Agilent Technologies, Böblingen, Germany). Primers are listed in the Supplementary table 1. Samples without enzyme in the reverse transcription reaction (Non-RT controls) were used as negative controls. Unspecific signals caused by primer dimers were excluded by non-template controls and by dissociation curve analysis^62^. β-Actin was used to normalize for the amounts of complementary DNA within each sample.

### Western blot analysis

The protein concentration of cell lysates was determined by amido black assays or Bradford protein assay (#5000001 BIO-RAD, Hercules, USA). Proteins were separated by sodium dodecyl sulfate–polyacrylamide gel electrophoresis (SDS-PAGE) and transferred to a polyvinylidene difluoride (PVDF) membrane. The membrane was incubated with antibodies against SHP2 (1:1000), pJAK2^Y1007/Y1008^ (1:500), JAK2 (1:1000) (# sc-7384, #sc-280, #sc-16566R, #sc-278, Santa Cruz Technologies, Heidelberg, Germany), pJAK2 (Y570) (1:500) (#CPA1629, Cohesion Biosciences), Collagen I (1:1000) (#ab138492, Abcam, Cambridge, UK), pSTAT3 (1:1000), STAT3 (1:1000) (#9145, #9139, Cell Signaling, Boston, USA) and horseradish peroxidase (HRP)-conjugated secondary antibodies (Dako, Hamburg, Germany). Blots were visualized by ECL. β-Actin was used as loading control. Western blots were quantified using the ImageJ Software (version 1.41). The uncropped scans of western blots presented in the figures are shown in Supplementary Fig. 5 and 6.

### Co-immunoprecipitation

After stimulation of fibroblasts with TGFβ (10 ng/ml) for 3 min, cells were collected in lysis buffer (Tris–HCl 50 mM, NaCl 150 mM, EDTA 1 mM, NP-40 1%, dithiothreitol (DTT) 1 mM and phenylmethylsulfonyl fluoride 1 mM). Five percent of the lysates were used as input. Then, 500 µg of protein lysate (Cell extract) was first incubated with 2 μg of antibodies against either SHP2 or serum IgG (all from Santa Cruz Biotechnology, Heidelberg, Germany) for 2 h at 4 °C under rotation. Subsequently, 30 μl of Protein A/G Sepharose was added to the samples. Unbound proteins were removed by washing with Tris buffer (Tris–HCl 50 mM, NaCl 150 mM, EDTA 1 mM, NP-40 1%). Sepharose-bound protein complexes were separated via SDS-PAGE followed by western blotting on a PVDF membrane. Proteins were visualized via ECL prime kit (GE Healthcare, Braunschweig, Germany).

### Phosphatase activity assay (PTP activity assay)

Protein samples were isolated from human fibroblasts and SHP2 immunprecipitated as described above. Para-nitrophenyl phosphate (p-NPP; Sigma-Aldrich) was used as enzyme substrate. The SHP2 immune complexes were washed three times in Tris buffer and once in phosphatase buffer (30 mM HEPES pH 7.4, 120 mM NaCl). Afterwards, samples were resuspended with 200 µl of phosphatase assay buffer (30 mM HEPES pH 7.4, 120 mM NaCl, 5 mM p-NPP, 1 mM DTT and 65 ng/µl bovine serum albumin) and incubated at 30 °C for 30 to 90 min. Hydrolysis of p-NPP was determined by reading the absorbance at 405 nm with a microtiter plate reader (spectrophotometer)^20^. A Recombinant Human SHP2 (R&D Systems, Minneapolis, USA) was used to generate a standard curve (0, 1, 2, 4, 6, 8 and 10 ng).

### Quantification of collagen protein

The amount of soluble collagen in cell culture supernatants was quantified using the SirCol collagen assay (Biocolor, Belfast, Northern Ireland). The total collagen content of tissue samples was determined by hydroxyproline assays using the chloramines-T method^28,63^. In brief, samples were digested with 6 M HCl for 4–6 h. Samples were centrifuged to remove debris and pH of the solution is adjusted to 7. Samples were hydrolyzed by incubation at 60 °C for 30 min. The cloramines-T was added to the hydrolyzate to allow oxidation followed by the addition of Ehrlich’s aldehyde reagent. The absorbance intensity of each sample was analyzed at 550 nm using a microtiter plate reader spectrophotometer.

### Immunohistochemistry and immunofluorescence staining

Formalin-fixed, paraffin-embedded skin sections or fibroblasts fixed in 4% paraformaldehyde and permeabilized by 0.25% Triton X-100 were stained with antibodies against α-SMA (1:1000) (Life Technologies), SHP2 (1:200) (#sc-280, Santa Cruz Technologies, Heidelberg, Germany), P4Hβ (1:200) (#AP08767PU-N, Acris Antibodies, Herford, Germany), vimentin (1:500) (#20346, Abcam), CD31 (1:200) (#AF3628, R&D Systems, Minneapolis, USA) and CD45 (1:200) (#MA5-17687, Thermo Fisher, Massachusetts, USA). HRP-conjugated or Alexa Fluor antibodies (1:200) (Life Technologies) were used as secondary antibodies. Irrelevant isotype-matched antibodies served as controls. Stress fibers were visualized with rhodamine-conjugated phalloidin (#R415, Sigma-Aldrich). Nuclei were counterstained using 4′,6-diamidino-2-phenylindole (DAPI; Santa Cruz Biotechnology). The staining was analyzed using a Nikon Eclipse 80i microscope (Nikon, Badhoevedorp, Netherlands). Voronoi tessellation of in vivo immunofluorescence pictures were performed using the ImageJ2 software^64,65^.

### Plasmid and reporter constructs

pJ3-SHP2^C459S^ and pJ3-SHP2^WT^ were provided by Ben Neel^66^ via Addgene (Cambridge, USA, plasmids #8319 and #8317, respectively). The Luciferase reporter plasmids p-TA-luc and pSTAT3-TA-luc were purchased from ClonTech (Mountain View, CA, USA). Fibroblasts were transfected with 5 μg of either plasmid or empty control vectors using the Amaxa 4D-Nucleofector (Amaxa, Cologne, Germany). The transfection efficiency was determined by co-transfection with vectors encoding for *β-galactosidase* (Promega, Mannheim, Germany).

The plasmid pCMV3-Flag-JAK2 encoding the human *JAK2* was purchased from Sino Biological (Beijing, China). In vitro mutagenesis of JAK2 was performed using the QuickChange Multi site-directed mutagenesis kit (Agilent Technologies) to yield JAK2 ∆Y570F, a JAK2 mutant that cannot be phosphorylated at the inhibitory site Y570. After verification of the correct sequence, the construct was transfected in dermal human fibroblasts using Amaxa 4D-Nucleofector for overexpression studies.

### Histological analyses

Skin sections were stained with hematoxylin/eosin or trichrome. The dermal thickness was analyzed at four different sites in each mouse in a blinded manner^67^. Dermal thickness was analyzed with a Nikon Eclipse 80i microscope (Nikon) at 100-fold magnification by measuring the distance between the epidermal–dermal junction and the dermal–subcutaneous fat junction at sites of induration at three consecutive skin sections of each animal^68^. For direct visualization of collagen fibers, Sirius Red staining was performed (Sigma-Aldrich).

### Statistics

All data are presented as median ± s.e.m, and differences between the groups were tested for their statistical significance by Mann–Whitney *U*-test. The *p* values of less than 0.05 were considered as statistically significant; *p* values are expressed as follows: 0.05 > *p* > 0.01*; 0.01 > *p* > 0.001**; *p* < 0.001***. GraphPad Prism software 7.0 was used for statistical analysis. The sample size was estimated based on previous experiments. No statistical method was used to predetermine sample size.

Experiments were not done in a blinded fashion except when specifically indicated. There were no exclusion criteria for the human and animal experiments. Mice were stratified according to sex and then randomized into the different treatment groups. Cells from human donors were also randomized.

### Data availability

The datasets generated and analyzed during the current study are available from the corresponding authors on reasonable request.

## Electronic supplementary material

Supplementary Information
