# Supplementary material for: The tyrosine phosphatase SHP2 controls TGFβ-induced STAT3 signaling to regulate fibroblast activation and fibrosis
Source: Nat Commun. 2018 Aug 14;9:3259. doi: 10.1038/s41467-018-05768-3 (PMC6092362; doi:10.1038/s41467-018-05768-3)
Supplement: Supplementary file 1 — Supplementary Information [file 41467_2018_5768_MOESM1_ESM.pdf]

# Supplementary Information:

# The tyrosine phosphatase SHP2 controls TGFβ-induced STAT3 signaling to regulate fibroblast activation and fibrosis

**Zehender *et al.***

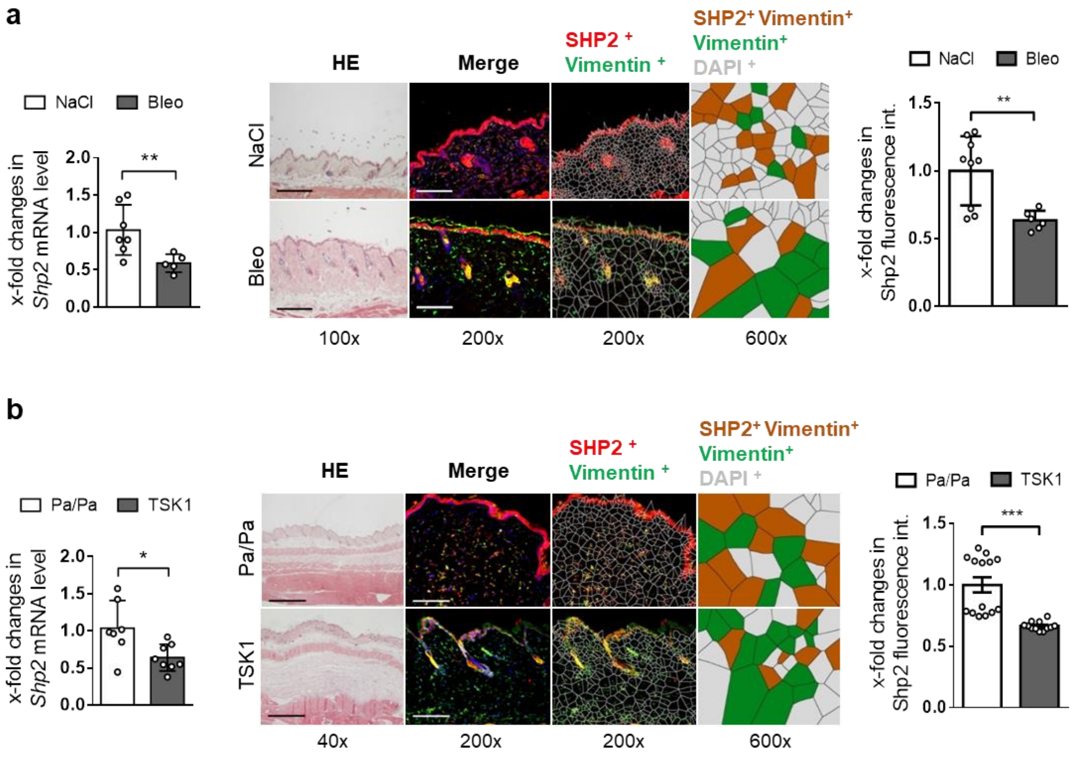


**Supplementary Figure 1: Decreased expression of SHP2 in experimental skin fibrosis. a:** Decreased expression of Shp2 and reduced mRNA levels of *Shp2* in the skin of mice challenged with bleomycin (n ≥ 5). Representative HE images and immunofluorescence staining of SHP2, vimentin and DAPI shown at 200- and 600-fold magnification. Horizontal scale bar, 500 µm. Voronoi tessellated pictures and histograms of respective Immunofluorescence signals are included. **b:** Reduced mRNA levels of *Shp2* in TSK1 mice (2 mg tamoxifen over 5 days) (n ≥ 7). Representative images stained for SHP2, vimentin and DAPI shown at 200- and 600-fold magnification. Results shown are representative of a minimum of two independent experiments per model. All data are presented as median ± s.e.m. The p-values are expressed as follows: 0.05 > p > 0.01 as *; 0.01 > p > 0.001 as **; p < 0.001 as ***; ns= not significant. Significance was determined by Mann–Whitney test. NSC-87877= SHP1/SHP2 inhibitor; TSK1= Tight skin; Pa/Pa= Tight skin control; Bleo= Bleomycin**.**

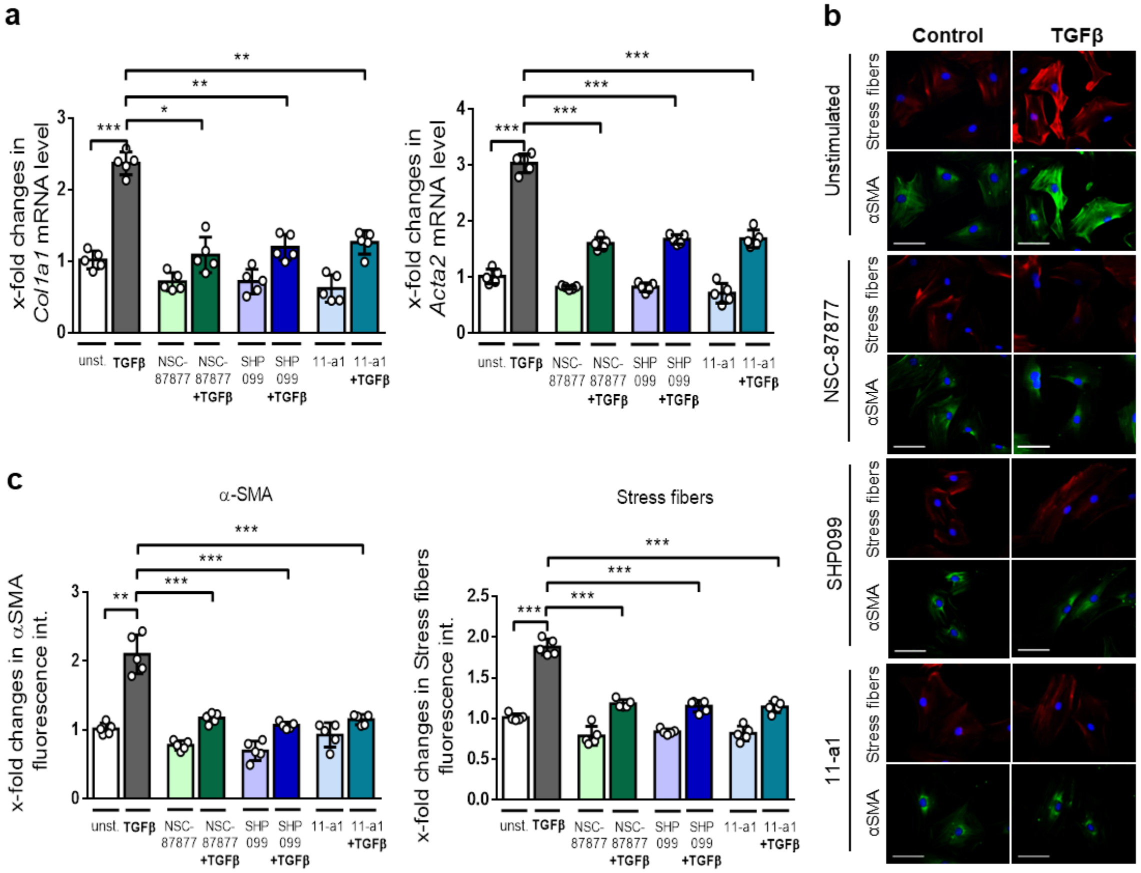


**Supplementary Figure 2: Inhibition of Shp2 by NSC-87877 (100 µM), SHP099 (1.4 µM) and 11-a1 (0.2 µM) inhibitors in cultured dermal fibroblasts. a:** mRNA levels of *Col1a1* and *Acta2* (n = 5). **b-c:** Representative immunofluorescence staining of αSMA, Stress fibers and DAPI shown at 400-fold magnification (**b**) with quantification (**c**). Horizontal scale bar, 500 µm. Results shown are representative of three independent experiments. All data are presented as median ± s.e.m. The p-values are expressed as follows: 0.05 > p > 0.01 as *; 0.01 > p > 0.001 as **; p < 0.001 as ***; ns= not significant. Significance was determined by Mann–Whitney test. NSC-87877, SHP099 and 11-a1 are SHP1/SHP2 inhibitor; unst.= unstimulated.

a

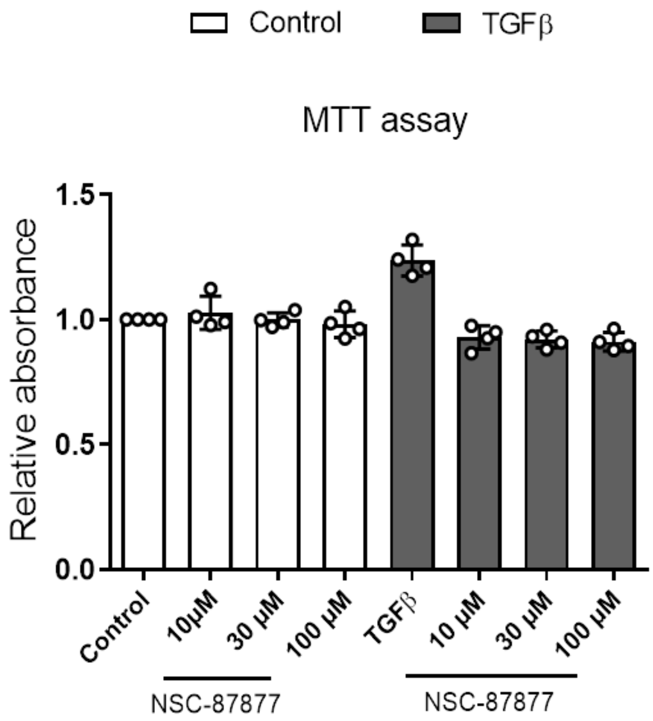


**Supplementary Figure 3: a:** Treatment with NSC-87877 does not modify the metabolic activity in resting fibroblasts, but prevents the increase induced by TGFβ. Results shown are representative of two independent experiments (n = 4). All data are presented as median ± s.e.m. The p-values are expressed as follows: 0.05 > p > 0.01 as *; 0.01 > p > 0.001 as **; p < 0.001 as ***; ns= not significant. Significance was determined by Mann–Whitney test. NSC-87877= SHP1/SHP2 inhibitor; MTT= tetrazolium dye.

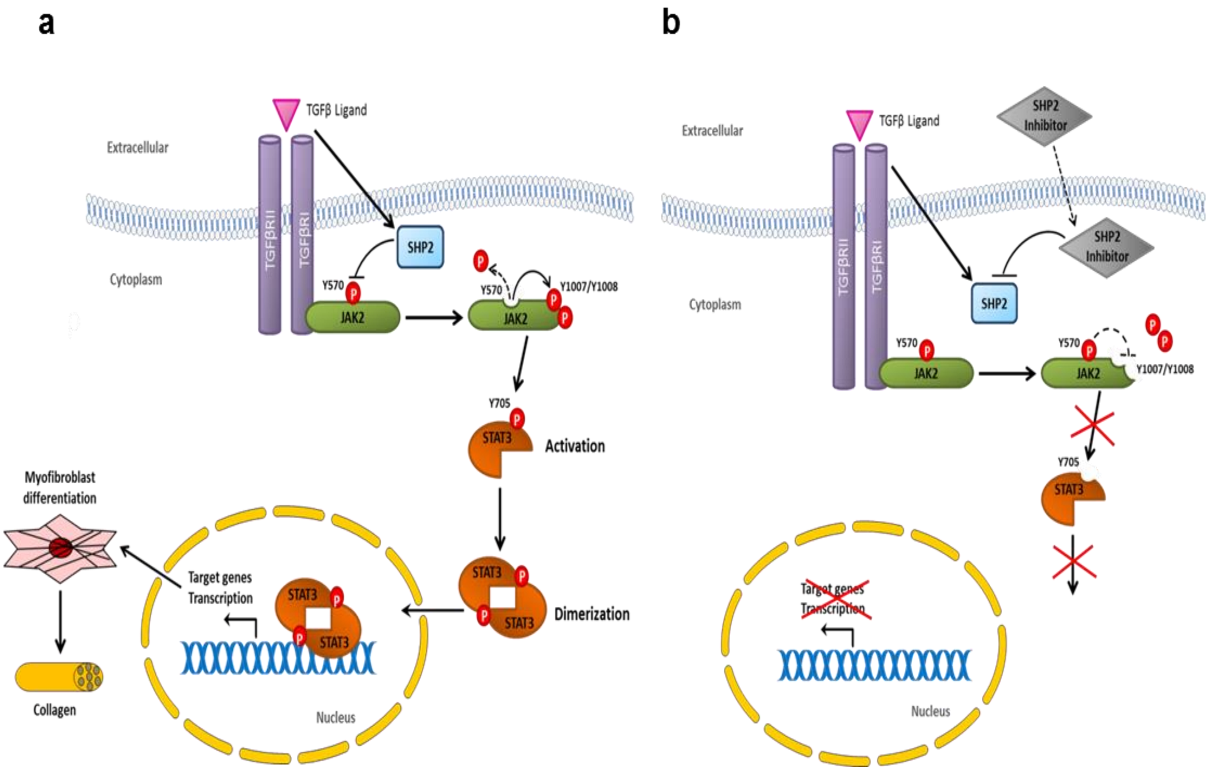


**Supplementary Figure 4:** Schematic summary of the proposed role of SHP2 in TGFβ-dependent fibroblast activation **(a)** and of the effects of SHP2-inhibitors **(b).** The Figure was created by the authors using in part images from Servier Medical Art, licensed under a Creative Common Attribution 3.0 Generic License. http://smart.servier.com/. P= Phosphorylation

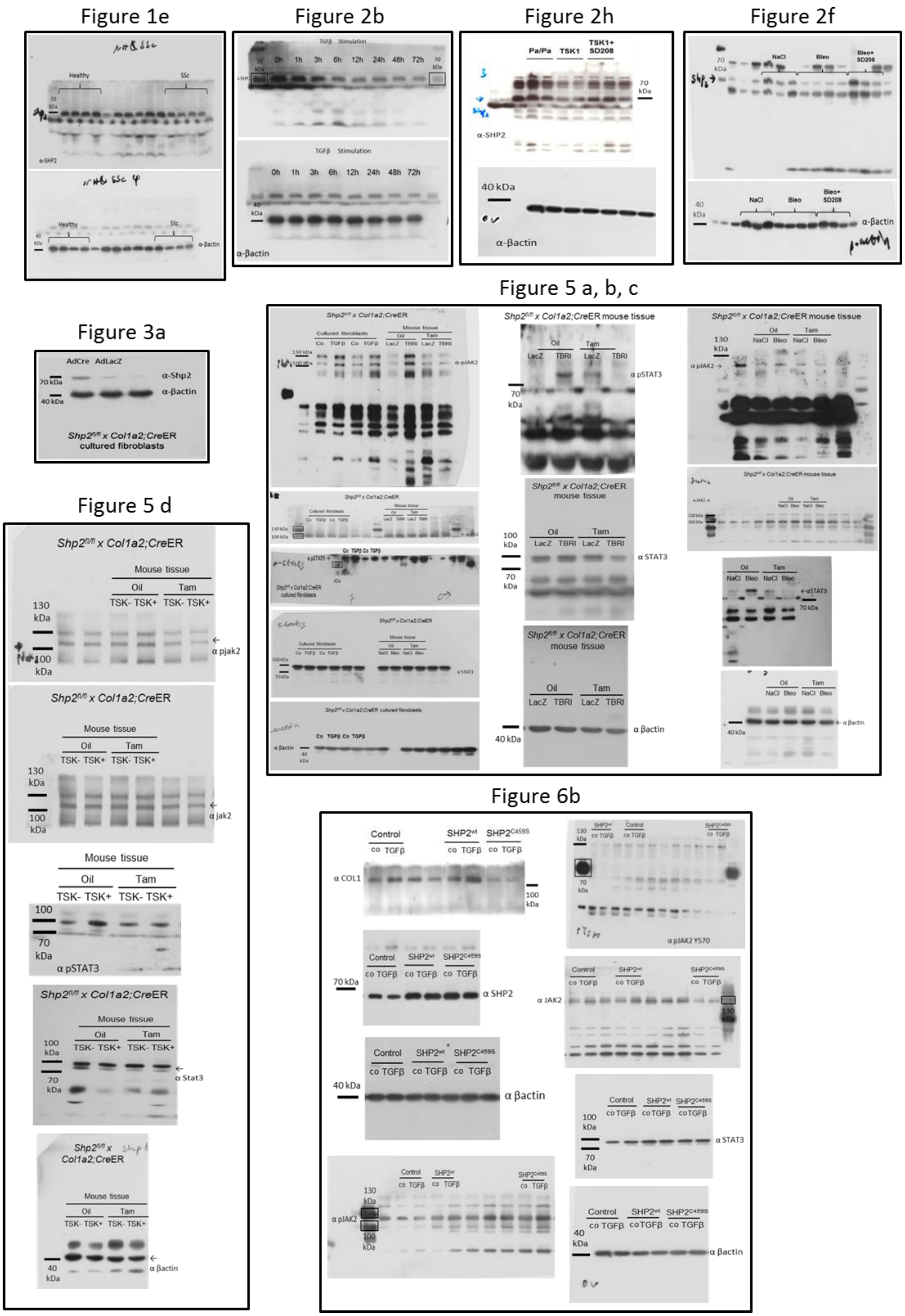


**Supplementary Figure 5:** Uncropped scan of Original Western blots.

Figure 7e

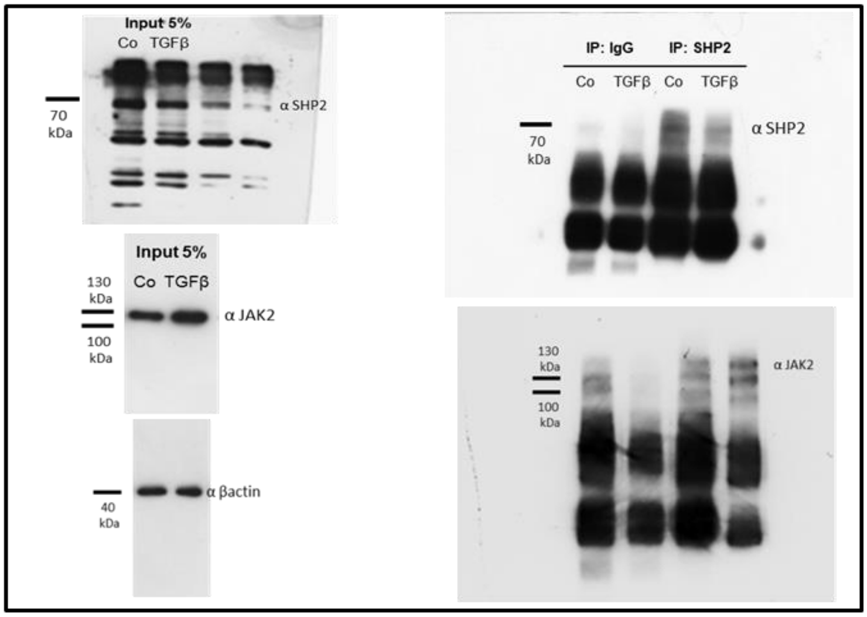


Figure 8d

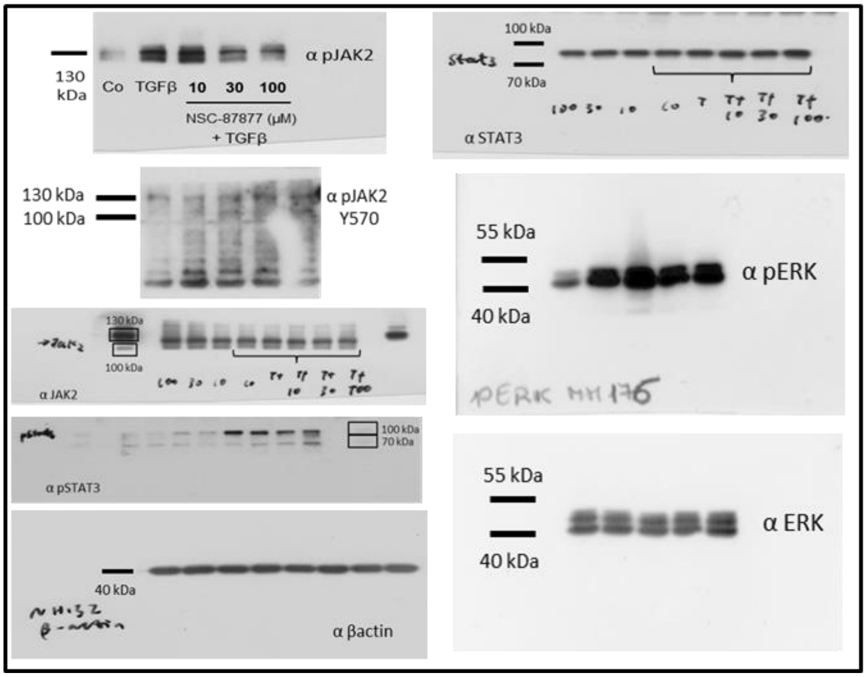


**Supplementary Figure 6:** Uncropped scan of Original Western blots.

**Supplementary Table 1:** primers

| | |
|---|---|
| human β-ACTIN forward | 5’-AGA AAA TCT GGC ACC ACA CC-3’ |
| human β-ACTIN reverse | 5’-TAG CAC AGC CTG GAT AGC AA-3’ |
| human SHP2 forward | 5’-TAT CCT CTG AAC TGT GCA GAT CC-3’ |
| human SHP2 reverse | 5’-TCT GGC TCT CTC GTA CAA GAA AA-3’ |
| human COL1A1 forward | 5’-ACG AAG ACA TCC CAC CAA TC-3’ |
| human COL1A1 reverse | 5’- ATG GTA CCT GAG GCC GTT C-3’ |
| human ACTA2 forward | 5’-TGG GCT GAA GCG CAC TGA CC-3’ |
| human ACTA2 reverse | 5’- CCG CGG CTC TTG CCC ACA T-3’ |
| murine β-actin forward | 5’-TCT TTG ATG TCA CGC ACG AT-3’ |
| murine β-actin reverse | 5’-TAC AGC TTC ACC ACC ACA-3’ |
| murine shp2 forward | 5’-GGA GAG CAT CGT GGA TGC-3’ |
| murine shp2 reverse | 5’-TCC CAG CGC TGC AGT GAA-3’ |
| murine Acta2 forward | 5’-ATG CCT CTG GAC GTA CAA CTG-3’ |
| murine Acta2 reverse | 5’-CAC ACC ATC TCC AGA GTC CA-3’ |
